# General practitioner use of a C-reactive protein point-of-care test to help target antibiotic prescribing in patients with acute exacerbations of chronic obstructive pulmonary disease (the PACE study): study protocol for a randomised controlled trial

**DOI:** 10.1186/s13063-017-2144-8

**Published:** 2017-09-29

**Authors:** Janine Bates, Nick A. Francis, Patrick White, David Gillespie, Emma Thomas-Jones, Rachel Breen, Nigel Kirby, Kerry Hood, Micaela Gal, Rhiannon Phillips, Gurudutt Naik, Jochen Cals, Carl Llor, Hasse Melbye, Mandy Wootton, Evgenia Riga, Ann Cochrane, Robin Howe, Deborah Fitzsimmons, Bernadette Sewell, Mohammed Fasihul Alam, Christopher C. Butler

**Affiliations:** 1 South East Wales Trials Unit, Centre for Trials Research, Cardiff University, Neuadd Meirionnydd, Heath Park, Cardiff, CF14 4XW UK; 2 Department of Population Medicine, Cardiff University School of Medicine, 5th Floor, Neuadd Meirionnydd, Heath Park, Cardiff, CF14 4YS UK; 3 Department of Primary Care and Public Health Sciences, King’s College London, London, SE1 3QD UK; 4 Department of General Practice, Care and Public Health Research Institute, Maastricht University, Maastricht, Netherlands; 5 University Institute in Primary Care Research Jordi Gol, Primary Healthcare Centre Via Roma, Barcelona, c. Manso, 19, 3rd floor, 08015 Barcelona, Spain; 6 Institute of Community Medicine, University of Tromsø, Tromsø, Norway; 7 Specialist Antimicrobial Chemotherapy Unit, University Hospital of Wales, Heath Park, Cardiff, CF14 4XW UK; 8 The Nuffield Department of Primary Care Health Sciences, University of Oxford, Radcliffe Observatory Quarter, Woodstock Road, Oxford, OX2 6GG UK; 9 Swansea Centre for Health Economics, College of Human and Health Sciences, Room 216, Vivian Tower, Swansea University, Singleton Park, Swansea, SA2 8PP UK; 10 Public Health Department, College of Health Sciences, Qatar University, PO Box 2713, Doha, Qatar

**Keywords:** Acute exacerbation, Chronic obstructive pulmonary disease, Primary care, Point-of-care test, C-reactive protein (CRP), Near-patient testing, Rationalising antibiotic prescribing, Antibiotic resistance, Cost-effectiveness, Resistance

## Abstract

**Background:**

Most patients presenting with acute exacerbations of chronic obstructive pulmonary disease (AECOPD) in primary care are prescribed an antibiotic, which may not always be appropriate and may cause harm. C-reactive protein (CRP) is an acute-phase biomarker that can be rapidly measured at the point of care and may predict benefit from antibiotic treatment in AECOPD. It is not clear whether the addition of a CRP point-of-care test (POCT) to clinical assessment leads to a reduction in antibiotic consumption without having a negative impact on COPD health status.

**Methods/design:**

This is a multicentre, individually randomised controlled trial (RCT) aiming to include 650 participants with a diagnosis of AECOPD in primary care. Participants will be randomised to be managed according to usual care (control) or with the addition of a CRP POCT to guide antibiotic prescribing. Antibiotic consumption for AECOPD within 4 weeks post randomisation and COPD health status (total score) measured by the Clinical COPD Questionnaire (CCQ) at 2 weeks post randomisation will be co-primary outcomes. Primary analysis (by intention-to-treat) will determine differences in antibiotic consumption for superiority and COPD health status for non-inferiority. Secondary outcomes include: COPD health status, CCQ domain scores, use of other COPD treatments (weeks 1, 2 and 4), EQ-5D utility scores (weeks 1, 2 and 4 and month 6), disease-specific, health-related quality of life (HRQoL) at 6 months, all-cause antibiotic consumption (antibiotic use for any condition) during first 4 weeks post randomisation, total antibiotic consumption (number of days during first 4 weeks of antibiotic consumed for AECOPD/any reason), antibiotic prescribing at the index consultation and during following 4 weeks, adverse effects over the first 4 weeks, incidence of pneumonia (weeks 4 and 6 months), health care resource use and cost comparison over the 6 months following randomisation. Prevalence and resistance profiles of bacteria will be assessed using throat and sputum samples collected at baseline and 4-week follow-up. A health economic evaluation and qualitative process evaluation will be carried out.

**Discussion:**

If shown to be effective (i.e. leads to a reduction in antibiotic use with no worse COPD health status), the use of the CRP POCT could lead to better outcomes for patients with AECOPD and help reduce selective pressures driving the development of antimicrobial resistance. PACE will be one of the first studies to evaluate the cost-effectiveness of a POCT biomarker to guide clinical decision-making in primary care on patient-reported outcomes, antibiotic prescribing and antibiotic resistance for AECOPD.

**Trial registration:**

ISRCTN registry, ID: ISRCTN24346473. Registered on 20 August 2014.

**Electronic supplementary material:**

The online version of this article (doi:10.1186/s13063-017-2144-8) contains supplementary material, which is available to authorized users.

## Background

Point-of-care tests (POCTs) for acute infections are being promoted to reduce inappropriate prescribing, reduce antimicrobial resistance and to improve patient-reported outcomes [1–4]. While POCTs are frequently subjected to evaluations of analytic performance, they have often been introduced into routine care before determining their clinical effectiveness via rigorous clinical trials and without understanding cost-effectiveness using relevant health and service delivery outcomes.

Better targeting of antibiotics in acute exacerbations of chronic obstructive pulmonary disease (AECOPD) represents a major opportunity for antimicrobial stewardship and improved patient care. Over 80% of all antibiotics are prescribed in the community [5] with high prescribing of broad-spectrum antibiotics a particular concern. AECOPD accounts for over two million antibiotic prescriptions each year in the UK [6]. Cohort studies of patients recruited in secondary care (which may not be representative of primary care but is the best relevant data available) suggest that most COPD patients in the UK will suffer between 2.5 and 3 AECOPD per year. Over 70% of patients presenting with AECOPD in primary care are prescribed an antibiotic, accounting for 4.6% of all antibacterial prescriptions every year [7] . COPD patients are an important and increasingly large group who are at risk of significant mortality, morbidity and hospitalisation and, as such, are more likely to be prescribed broad-spectrum antibiotics [8]. However, many AECOPD are triggered by non-bacterial causes, such as viral infections and environmental factors including common pollutants or weather. It has been estimated that approximately 70% of AECOPD are triggered by an infection and 30% are caused by environmental factors. Of the 70% that are triggered by an infection, potential pathogenic bacteria are only isolated in 20–58% of clinical samples, while pathogenic respiratory viruses can be detected in approximately 50% [9–11].

Current antibiotic prescribing recommendations for general practitioners (GPs) are generally based on symptoms alone (Anthonisen criteria; [12, 13]). However, these symptoms are subjective and have insufficient diagnostic accuracy to predict which patients can safely be managed without antibiotics. Both our placebo-controlled trial of antibiotics for AECOPD in primary care [14] and systematic reviews [15] suggest that many patients with AECOPD in primary care do not benefit from antibiotic treatment. Overuse of antibiotics drives antimicrobial resistance [16] and is facilitated by the unnecessary consumption of antibiotics for COPD. Antimicrobial treatment in patients with COPD decreases the infecting load but does not usually entirely eradicate organisms in the airways, increasing the risk of resistant bacteria in COPD patients [17]. Infections with antibiotic-resistant *Streptococcus. pneumonia*e in patients with COPD are associated with antibiotic exposure [18, 19]. A meta-analysis of seven studies of respiratory tract bacteria that included 2605 participants showed that the pooled odds ratio (OR) for resistance was 2.4 (1.4 to 3.9) within 2 months of antibiotic treatment, and 2.4 (1.3 to 4.5) within 12 months [5]. The unnecessary use of antibiotics for AECOPD not only contributes to the increasingly pressing public health threat of antibiotic resistance, it also poses a risk for the individual, and may increase the risk of subsequent antibiotic-resistant exacerbations and hasten disease progression. Indiscriminate use of antibiotics in patients with COPD is particularly high risk because the respiratory tracts of those affected are frequently colonised with potential pathogens [20]. Unnecessary antibiotics also increase the risk of patient side effects, waste money, and undermine self-care [21].

A Cochrane systematic review of the use of antibiotics in the management of exacerbations of COPD included 16 trials (*n* = 2068 participants), and reported that there was insufficient evidence of effectiveness to guide antibiotic prescribing decisions in primary care [15].

C-reactive protein (CRP) is an acute-phase protein found in the blood. The serum level of CRP increases rapidly during infections, particularly in severe bacterial infections. A prospective evaluation of 36 biomarkers found that CRP was the most selective biomarker to confirm AECOPD, and in combination with Anthonisen criteria produced an area under the curve (AUC) of 0.88 (95% confidence interval (CI) 0.82–0.93), indicating that it had good diagnostic accuracy [22]. High serum CRP is correlated with sputum purulence and raised serum leucocyte counts and serum CRP is higher in the presence of bacterial infection [23, 24]. CRP rises in patients with AECOPD and is correlated with Anthonisen score and the degree of airflow limitation in hospitalised patients [25, 26]. As CRP levels are especially raised in the presence of bacterial infection the treatment effect of antibiotics increase with higher values of CRP [27]. A CRP value above 50 mg/L (mean CRP of 97 mg/L, 95% CI 49–145) in hospitalised patients with AECOPD is associated with pneumonia and such patients are likely to benefit from antibiotics [26]. CRP measurement independently distinguished between pneumonia and other causes of exacerbations in another study of patients hospitalised with asthma and COPD (cut-off value of 48 mg/L with sensitivity of 91% and specificity of 93%) [28]. In a randomised controlled trial we conducted in patients with AECOPD in primary care, we found no difference in clinical cure between antibiotics and placebo in those with a CRP < 40 (risk ratio (RR) for clinical failure = 0.72 (95% CI 0.28–1.82) *p* = 0.484) [23].

In an as yet unpublished study we found that over 50% of COPD patients experiencing an exacerbation had a CRP < 8 mg/L [29]. Our recent placebo-controlled trial of antibiotics for AECOPD in primary care found marginal benefit from antibiotic treatment in patients with only one or two Anthonisen criteria. Using Anthonisen criteria to predict benefit from antibiotic treatment produced an area under the curve (AUC) of 0.708 (95% CI 0.616–0.801). Adding CRP increased this to an AUC of 0.842 (95% CI 0.76–0.924). Based on these data we anticipate that using a CRP test alongside clinical assessment will make it possible to safely reduce the antibiotic prescription rate for this condition to around 45%.

CRP POCTs are widely available and are already commonly used to help guide antibiotic prescribing decisions, including for lower respiratory tract infections (LRTIs) and AECOPD in primary care in a number of European countries (mostly Scandinavian) [30]. In two trials evaluating the use of a CRP POCT to help target antibiotic treatment for LRTIs in primary care [31, 32], antibiotics were prescribed to 68% and 48% in the usual care groups, respectively, and to 39% and 33% of patients managed by clinicians using a CRP POCT (with training). CRP POCT was cost-effective in reducing antibiotic prescribing for LRTIs when there are no tests, or low willingness to pay for the tests [31, 33]. However, the benefits of CRP POCT in conjunction with clinical examination has not yet been evaluated for AECOPD in primary care in a randomised controlled trial. Now that better and more rapid CRP POCTs are available [34], there is potential for this technology to be widely used for a variety of acute infections in primary care to better guide antibiotic prescribing and in doing so, help reduce unnecessary antibiotic consumption and thus contain antibiotic resistance. PACE seeks to establish whether a CRP POCT can safely and cost-effectively be used to better target antibiotic treatment for AECOPD in primary care to those that are most likely to benefit, so that overall antibiotic use is decreased without compromising COPD-related health status.

## Methods/design

### Objectives

The primary objective is to determine whether the addition of a CRP POCT (with training on test use and advice on interpretation) to usual care for managing AECOPD leads to a reduction in antibiotic consumption for AECOPD without negatively impacting on COPD health status, compared with usual care alone. Table 1 lists the primary and secondary objectives and outcome measures.

**Table 1:** Primary and secondary objectives and outcome measures

	Objectives	Outcome measures	Time point(s) of evaluation of this outcome measure
Primary	To determine whether the addition of a CRP POCT (with training on test use and advice on interpretation) to usual care for managing AECOPD leads to a reduction in antibiotic consumption for AECOPD compared to usual care alone	Antibiotic consumption (any consumption of antibiotics for AECOPD vs. no consumption of antibiotics for AECOPD)	First 4 weeks post randomisation
Primary	To determine whether the addition of a CRP POCT (with training on test use and advice on interpretation) to usual care for managing AECOPD leads to a reduction in antibiotic consumption for AECOPD without negatively impacting on COPD health status compared to usual care alone	Recovery in terms of COPD health status as assessed using the Clinical COPD Questionnaire (CCQ) total scores	2 weeks post randomisation
Secondary	To assess the effect of using a CRP POCT for AECOPD in primary care on:	Prevalence of potentially pathogenic bacteria (including *S. pneumoniae*, *Haemophilus* spp. and *Enterobacteriacae*) cultured from sputum at 4 weeks and the proportion of bacteria that are resistant	4 weeks post-randomisation
		Prevalence of commensal organisms cultured from throat swabs at 4 weeks and proportion of bacteria that are resistant	4 weeks post randomisation
		COPD health status over time measured using the CCQ total score	At weeks 1, 2 and 4 post randomisation
		CCQ symptoms domain	At weeks 1, 2 and 4 post randomisation
		CCQ function state domain	At weeks 1, 2 and 4 post randomisation
		CCQ mental state domain	At weeks 1, 2 and 4 post randomisation
		Total antibiotic consumption (number of days antibiotics consumed for AECOPD/any reason)	First 4 weeks post randomisation
		Health utility measured using the EuroQol-5D (EQ-5D)	At weeks 1, 2 and 4 and at month 6 post randomisation
		All-cause antibiotic consumption	During the first 4 weeks post randomisation
		Antibiotic prescribing	At the index consultation
		Antibiotic prescribing	During the first 4 weeks post randomisation
		Use of other COPD treatments including orally administered steroids	During the first 4 weeks post randomisation
		Adverse effects potentially attributable to antibiotics prescribed for the exacerbation	During the first 4 weeks post randomisation
		Primary and secondary care consultations, including hospitalisations	At week 4 and month 6
		Costs (total NHS cost) and cost-effectiveness	At month 6
		Incidence of pneumonia (measured by patient and GP report)	At week 4 and month 6
		Disease-specific, health-related quality of life over time measured using the CRQ-SAS (dyspnoea, fatigue, emotion function, mastery and total scores)	At month 6

*AECOPD* acute exacerbation of chronic obstructive pulmonary disease, *CCQ* Clinical COPD Questionnaire, *CRP* C-reactive protein point-of-care test, *POCT* point-of-care test, *CRQ-SAS* Chronic Respiratory Disease Questionnaire, self-administered, standardised, *GP* general practitioner

### Trial design

PACE is a two-arm, open, individually randomised (1:1) controlled trial involving general practitioners in general medical practices that are part of primary care research networks in the UK. Patients presenting with AECOPD will be randomised to clinical management based on usual care alone or usual care with the addition of a CRP POCT. Training in POCT use and interpretation will help to guide decisions about the use of antibiotic treatment for AECOPD.

Our primary research question centres on whether CRP POCT-informed management of patients with AECOPD reduces antibiotic use without negatively impacting on COPD health status. We will answer this question in terms of co-primary outcome measures; antibiotic consumption for AECOPD within 4 weeks post randomisation and COPD health status 2 weeks post randomisation. Between-group differences in antibiotic consumption will be investigated for superiority, while differences in COPD health status will be investigated for non-inferiority.

### Outcome measures

The co-primary outcome measure will comprise:

1. Antibiotic consumption at any point during the 4 weeks post randomisation for AECOPD, measured using telephone interviews at 1 week and 2 weeks and face-to-face interview at 4 weeks
2. COPD health status measured by the Clinical COPD Questionnaire (CCQ)[35] via telephone interview at 2 weeks. The CCQ is a patient-centred health status measure that has been well validated and is widely used in patients with COPD [36]

A 4-week time window was selected for the antibiotic consumption outcome in order to measure the consumption of antibiotics prescribed at the initial consultation in addition to those that are related to the AECOPD in question, but are initiated or prescribed at a later date. The CCQ outcome is measured at 2 weeks as this is the time when most patients will have recovered and, therefore, the point at which a difference would be most indicative of a delayed recovery.

Secondary outcome measures are listed in Table 1.

### Setting and timing

The first winter period will comprise an internal pilot in 10–15 general practices in Wales (2014 to 2015). Following the pilot, we aim to have at least 70 general practices in the UK that are open to recruitment during the winters of 2015-2016 and 2016-2017.

### Trial intervention

PACE will assess the use of a CRP POCT to guide antibiotic treatment decisions for patients presenting in primary care with AECOPD. Patients randomised to the intervention arm will have a CRP POCT at every consultation for AECOPD that occurs in the 4 weeks following randomisation. Control patients will not have a CRP POCT (as part of this study) at any time during their participation.

The CRP POCT Afinion device (Alere Inc.) and CRP cartridges will be offered to practices implementing the study (http://www.alere.com/en/home/product-details/afinion-crp.html). This POCT requires 1.5 μl of capillary blood (finger prick) and takes less than 4 min to provide a quantitative result. Other validated, CE-marked devices and CRP cartridges giving a quantitative result within the range of the Afinion POCT, and requiring a similar volume of blood from a finger prick, will also be eligible for use in the study where a practice prefers to use CRP POCTs that they might already be using.

The prescribing clinician will use the results of the CRP POCT to help guide their antibiotic prescribing decision when consenting patients are randomised to care with the addition of the CRP POCT. Participating clinicians (GPs, practice nurses and health care assistants) will be provided with study-specific training, which will include guidance, for the clinical prescribers, on interpreting CRP results in the context of AECOPD (Table 2).

**Table 2:** Guidance on interpreting C-reactive protein (CRP) results

CRP guidance:	
The decision to prescribe antibiotics or not has to be based on a comprehensive assessment of the likely risks and benefits given: • The patient’s underlying health status (chronic obstructive pulmonary disease (COPD) severity, comorbidities, frailty) • Clinical features of the current exacerbation	
Measurement of CRP can aid decision-making but is not meant to replace clinical assessment. Patients with the following features are likely to be at increased risk of complications: • Severe COPD (GOLD grade III) • Past history of severe exacerbations (requiring hospitalisation) • Significant comorbidities (e.g. heart failure, poorly controlled diabetes, lung cancer)	
Sputum purulence is currently the best clinical predictor of bacterial infection. However: • Patient-reported sputum colour is generally not reliable • Purulence can be increased in viral infections as well as bacterial infections • Try and obtain a sputum sample in order to objectively assess sputum purulence where possible • Ask the patient how much the colour of their sputum has changed from its usual colour. This is particularly pertinent when it is not possible to objectively assess their sputum	
CRP measurement:	
CRP < 20	Antibiotics are unlikely to be beneficial and usually should not be prescribed
CRP 20–40	Antibiotics may be beneficial – mainly if purulent sputum is present. You may decide to prescribe antibiotics after taking into account the patient’s underlying health status and the features of the current exacerbation
CRP > 40	Antibiotics are likely to be beneficial. Consider prescribing antibiotics unless the patient is assessed as being at lower risk of complications and unlikely to have a bacterial infection (no increased sputum purulence and no features suggesting severe exacerbation)

### Control arm

Patients randomised to the control arm will receive usual clinical care. All participating sites will be provided with information on current best practice.

### Trial procedures

#### GP site selection and training

The study will be implemented in at least 70 general practices from five regional centres in the UK using existing National Institute for Health Research (NIHR) and Health and Care Research Wales infrastructure, supported by research professionals’ networks and a new Primary Care Incentive Scheme (Wales).

The trial team will monitor recruitment and, if necessary, close some practices and/or expand to new practices/regions.

All staff involved in trial-specific procedures (including recruitment/consent, collection of trial data, application of intervention and clinical assessments) will be appropriately trained in the relevant aspects of the procedures and in Good Clinical Practice (GCP) processes. This training will include providing an overview of the aims and rationale for the study, a summary of National Institute for Health and Care Excellence (NICE) and The Global Initiative for Chronic Obstructive Lung Disease (GOLD) guidance on managing AECOPD, and training in interpreting CRP POCT results (Table 2).

Representatives from Alere Inc. or the study team will provide general practices (who will be using an Afinion CRP machine) with specific training in using the CRP POCT, including quality-control procedures.

### Participant recruitment and consent

**Fig. 1:**
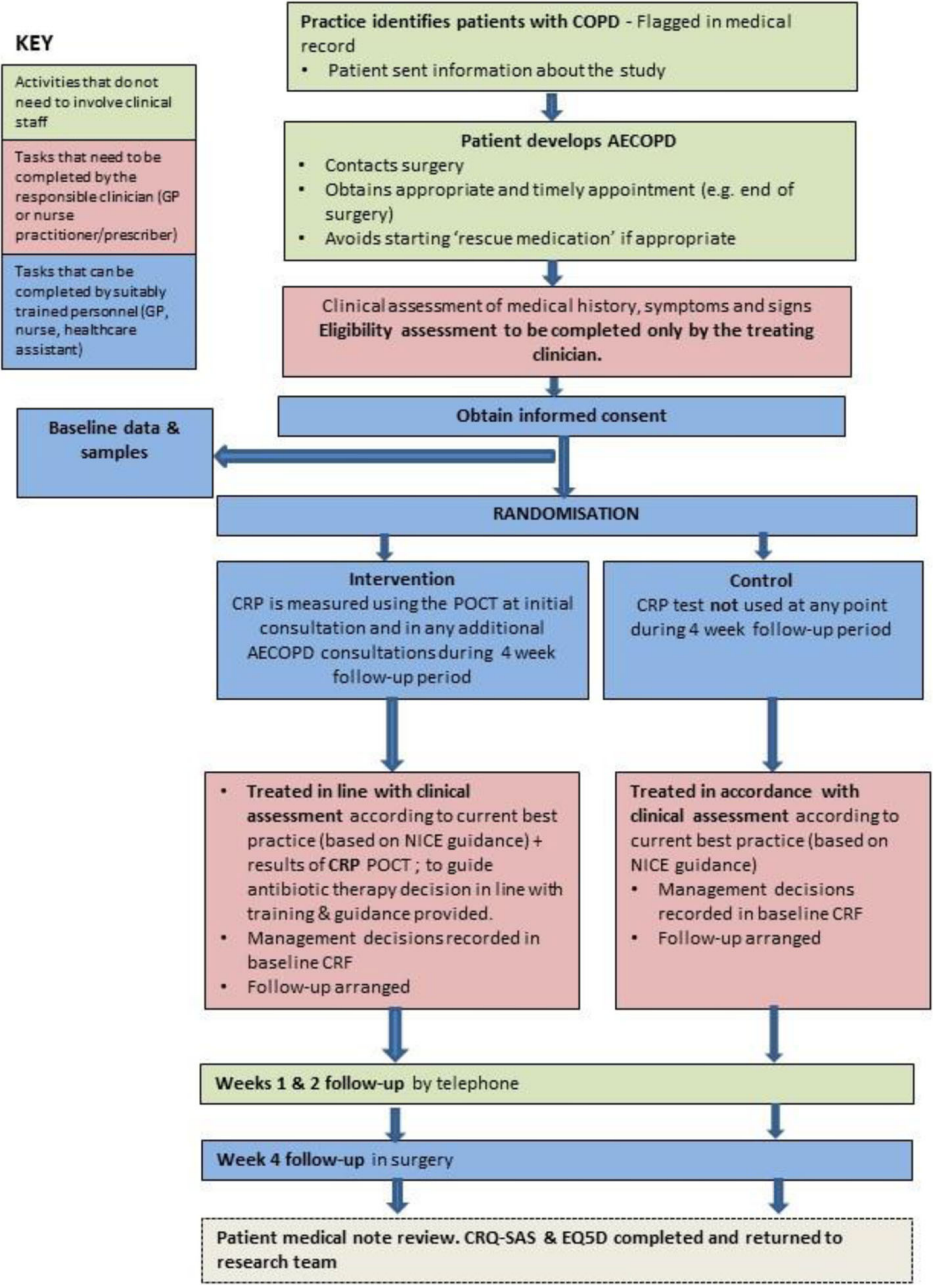
Participant flow diagram

The recruitment process is summarised in Fig. 1. Participating general practices will identify all potentially eligible patients at the start of the study usually from their existing COPD registers. Identified patients will be sent a letter, from their general practice, informing them about the study and a Participant Information Sheet (PIS). The letter will include informing the patient that if they are interested in taking part in the study and if they have home ‘rescue packs’ which contain antibiotics, that they should consider contacting their surgery for a timely appointment before deciding to start to taking their home supply of antibiotics.

### Participants

#### Assessment of participant eligibility:

A GP or nurse responsible for managing the patient’s current illness will complete the eligibility assessment.

Patients will be eligible for the trial if they meet the inclusion criteria and none of the exclusion criteria (Table 3).

**Table 3:** Eligibility criteria

Inclusion criteria	Exclusion criteria
Has a current acute exacerbation (presenting with at least 1 of the following: increased dyspnoea, increased sputum volume, increased sputum purulence) that has lasted for at least 24 h and no longer than 21 days	The responsible GP feels urgent referral to hospital is necessary
Diagnosis of COPD in clinical record/on COPD practice register	Severe illness (e.g. suspected pneumonia, tachypnoea > 30 breaths/min, respiratory failure)
Age 40 years or more	Concurrent infection at another site (e.g. UTI, cellulitis) that is likely to produce a systemic response
Able to provide informed consent	Past history of respiratory failure or mechanical ventilation
Patient should be able to provide the primary outcome data at 2 and 4 weeks within the expected windows	Currently taking antibiotics or has taken antibiotics for this acute exacerbation of COPD
	Active inflammatory condition (e.g. flare up of rheumatoid arthritis, gout or polymyalgia rheumatica)
	Has cystic fibrosis, a current tracheostomy or bronchiectasis
	Immunocompromised (e.g. AIDS, taking systemic immunosuppressive therapy or receiving anti-cancer radiotherapy or chemotherapy)
	Currently pregnant
	Previously been recruited into the PACE study

*COPD* chronic obstructive pulmonary disease, *GP* general practitioner, *UTI* urinary tract infection

Once consented to the study, participants will be allocated a unique trial number (participant ID), which will be the primary identifier for participants in the trial.

### Randomisation

Participants will be remotely randomised, following consent, using an online computerised randomisation system created by the Centre for Trials Research at Cardiff University. This will be operational 24 h a day. In addition, an 8.30 a.m. to 6.30 p.m. telephone back up will be available if the online system fails or the general practice has problems accessing the online site.

Participants will be randomised in a 1:1 ratio to receive either usual care alone (control) or usual care with the addition of CRP POCT (intervention). Randomisation will use minimisation, with a random element set at 80% to improve the integrity of the randomisation process. Anthonisen criteria (categorised as type 1, 2 or 3) will be used as a minimisation variable, so that balance is achieved with respect to differing levels of COPD exacerbation severity. Remote allocation will maintain allocation concealment from both the participant and the recruiting clinician up to the point of intervention, as this is an open study.

### Data collection

**Fig. 2:**
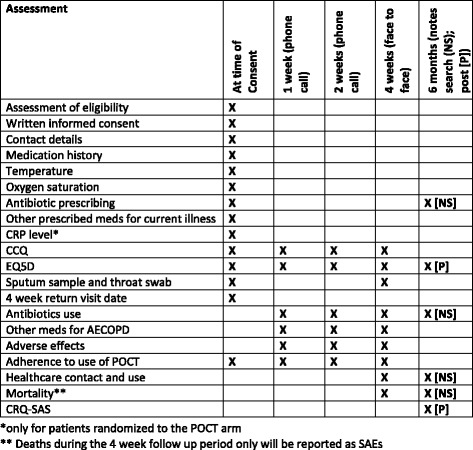
Schedule of assessments (SPIRIT figure)

In order to facilitate the process of patient recruitment and data collection into busy routine clinics, data collection can be conducted by any suitably trained clinician (nurse, health care assistant or GP). The timing and type of assessment (Standard Protocol Items: Recommendations for Interventional Trials (SPIRIT) Figure) is described in Fig. 2.

A SPIRIT Checklist is included as an Additional file (Additional file 1).

### Baseline appointment

The clinician carrying out the baseline appointment will record the number of days that the participant reports having symptoms from the acute exacerbation, their medical history, and clinical examination results (temperature, pulse, oxygen saturation and whether tachypnoea, crackles, wheezes, diminished vesicular sounds and evidence of consolidation).

A sputum sample (when participants are able to produce sputum) and throat swab samples (using charcoal swabs) will be obtained from the patient at the baseline appointment and recorded on the baseline Case Report Form (CRF). Recruiting clinicians will assess and record, on the baseline CRF, the colour of the participant’s sputum against a Bronkotest chart (Bronkotest Ltd.).

Participants will be asked to self-complete the baseline CCQ and the baseline European Quality of Life-5 dimensions (EQ-5D) questionnaire at this visit. The CRP test results will be recorded on the baseline CRF for those randomised to care with the addition of the CRP POCT.

Antibiotic prescribing and other management decisions will be recorded for all patients following randomisation, and after CRP testing for those randomised to the intervention.

### Follow-up data collection

The trial team will aim to telephone all participants at 1 week and 2 weeks post randomisation to collect information on their medication usage during that time period and also to obtain their responses to questions on the week-1 and week-2 CCQ and EQ-5D questionnaires. Participants will be invited to return to the surgery for a face-to-face consultation at 4 weeks post randomisation. The Chronic Respiratory Disease Questionnaire, self-administered, standardised (CRQ-SAS) and the EQ-5D will be posted to participants for completion and return at 6 months.

*Telephone calls* **–** *week 1* (time window of −1/+2 working days) and *week 2* (time window of −1/+7 working days).

Participants will be contacted by telephone by a member of the trial team. Participants will be given paper versions of the week-1 and-week 2 CCQ and EQ-5D questionnaires at their baseline visit and asked to complete them on each appropriate day (day 8 and day 15, with the baseline appointment being on day 1) prior to the telephone interview in order to facilitate data collection.

*Face-to-face visit* **–** *week 4* (time window of −3/+14 working days).

The week-4 face-to-face appointment will be arranged at the time of baseline assessment and appointments will be conducted by a member of the clinical team in the general practice or a clinical study officer/research nurse working for the local clinical research network at the general practice. The following data will be captured: medication consumption, adverse effects, time off paid work, diagnosis of pneumonia since the baseline appointment, any further CRP tests since the baseline appointment, health care consultations. Sputum (where possible) and throat swab samples will be obtained from the participant and the colour of the sputum assessed against a Bronkotest chart. Participants will be asked to self-complete the week-4 CCQ and the week-4 EQ-5D questionnaire at this visit. If a successful appointment does not take place at week 4, the study team will contact the participant by telephone to obtain a minimum dataset. This dataset includes antibiotic consumption during the third and fourth weeks after randomisation, health care resource use and completion of the CCQ and EQ-5D questionnaires. If the participant has missed their week-1 or week-2 follow-up telephone call, the site will be asked to carry out a minimum dataset with the participant at the week-4 appointment. This dataset captures information on the participants’ medication consumption.

### Collection of relevant data from electronic medical records at 6 months

Antibiotics prescribed in the 12 months prior to study inclusion, spirometry results and a full blood count result obtained prior to study inclusion will be recorded.

Spirometry results, prescriptions, and health service utilisation data will be recorded for each participant for the 6-month period following randomisation. General practices will be asked to provide these data on a proforma basis from a medical records review; alternatively, the data will be collected by a member of the trial team or clinical study officer/research nurse.

### Patient self-reported CRQ-SAS and EQ-5D – 6 months

Participants will be sent a copy of the CRQ-SAS and EQ-5D at 6 months post randomisation by post. Participants will be telephoned by the trial team 1 week after the due date to remind them to return the questionnaire by post or to offer to complete these instruments over the telephone at that point. If the questionnaire is not received within 1 week of the first telephone reminder, the trial team will telephone the participant again.

### Adverse events

Hospitalisation is expected within this patient population and will be collected and reported as part of routine follow-up. All other events fulfilling the definition of a serious adverse event (SAE), including death, that occur between the time of consent and the 4-week follow-up will be reported to the coordinating research centre within 24 h of the site becoming aware of the event.

### Microbiological assessment

Sputum (if available) and throat swab samples will be obtained at the recruitment visit (baseline) and at the face-to-face visit at week 4 and will be sent to the Specialist Antimicrobial Chemotherapy Unit (SACU), Public Health Wales at the University Hospital of Wales. Not all patients will be able to produce a sputum sample on request and, therefore, we would expect to have a lower return rate for these samples as compared to the throat swab samples at both baseline and week 4.

Sputum sample appearance (including colour and consistency) will be noted and all samples will be processed using the laboratory’s standard operating procedures. Potential pathogenic bacteria (including *S. pneumoniae*, *Haemophilus influenzae/parainfluenzae*, *Pseudomonas* spp., *Enterobacteriaceae* and *Staphylococcus aureus*) found in the sputum will be identified using the Matrix Assisted Laser Desorption Ionising Time of Flight Mass Spectrometry (MALDI-ToF-MS) and semiquantitative counts recorded. Susceptibilities will be performed on relevant bacterial species from sputum samples by disc diffusion using European Committee on Antimicrobial Susceptibility Testing (EUCAST) methodology and breakpoints. Throat swabs (charcoal) will be added to Tryptone soya broth and 50 μL spiral plated onto a range of non-selective plates and selective plates for identification purposes (e.g. Columbia blood agar, Isosensitest with blood) and antimicrobial-selective plates containing penicillin, third-generation cephalosporins, doxycycline, levofloxacin and clarithromycin at concentrations consistent with EUCAST breakpoints). Total bacterial counts of commensal organisms will be recorded on non-selective and selective agars; proportional quantification of resistant isolates will be determined from the selective media. All pathogens recovered, sputum samples and remaining broth from throat swabs will be stored at −80 °C.

### Process evaluation

A qualitative process evaluation will be embedded in the trial to provide a better understanding of the implementation and receipt of the intervention and the context in which it is delivered. This will aid interpretation of outcome results and possible uptake of the intervention should the trial have favourable benefits and risks [37]. Semistructured interviews will be carried out with up to 30 members of the participating primary care teams and up to 30 patients to gather in-depth information on their experience of participating in the study and their thoughts of management of AECOPD in primary care. These will be conducted by telephone. Approximately a third of these interviews will take place during the pilot phase of the trial, with the remainder being carried out during the full trial.

The objectives of the qualitative process evaluation are to:

1. Understand patient perspectives on the use of the CRP POCT to help guide the management of AECOPD
2. Understand clinician perspectives on the use of the CRP POCT to help guide the management of AECOPD
3. Understand barriers and facilitators to using the CRP POCT in primary care and to inform possible implementation and roll-out (if appropriate), including:
  - Views of primary care team members on conducting POCT testing for AECOPD in primary care
  - Patient perspectives on the routine management of AECOPD, including the use of antibiotics
  - Primary care clinicians’ views on the challenges involved in the routine management of AECOPD

An additional objective during the pilot phase will be to examine perceptions of the research processes to identify facilitators and barriers to participation so that these can be addressed as far as possible prior to the full trial phase.

Flexible topic guides will be used for the clinician and patient interviews covering themes relating to these objectives. The interviews will be audio-recorded and will be approximately 30 min in duration.

Patients who have agreed to be contacted for an interview will be telephoned by the research team within 2 weeks of their 4-week follow-up appointment with their GP (i.e. 4–6 weeks from their initial consultation). The trial team will write to participating practices to invite them to take part in the qualitative interviews, and this will be followed up by a telephone call to arrange an interview with a member of the primary care team.

Purposive sampling will be used to capture a range of views from health professionals and patients. During the internal pilot stage, approximately equal numbers of patients in each trial arm will be sampled to assess the acceptability of research procedures in each group. In the full trial, only patients from the CRP POCT arm will be interviewed. Patients will be sampled across geographical regions and will include patients who had, or had not, been prescribed antibiotics at their initial consultation. Similarly, general practices across all regions will be sampled. Interviews will be conducted with prescribing clinicians (i.e. GPs and nurse practitioners) and non-prescribing members of the primary care team who have been involved with the trial. In the internal pilot phase of the trial, recruitment of the primary care team will be associated with experience of the research processes (e.g. recruitment of patients), while in the full trial phase we will focus on members of the primary care team with experience of carrying out and/or interpreting results of the CRP POCT.

As part of a quantitative process evaluation, we will estimate adherence to the allocated treatment arm based on patient reports of receiving a finger-prick blood test and, where possible, verify and supplement these data from other sources, such as GP records and CSV files stored on the CRP Afinion machine.

## Analysis

### Statistical considerations

#### Sample size

We aim to have sufficient power to detect a 15% reduction from an estimated 70% that consume antibiotics for the AECOPD during the 4 weeks following randomisation [7]. Trials using CRP testing to reduce antibiotic prescribing for lower respiratory tract infection have resulted in absolute reductions in antibiotic prescribing in the region of 13–22% [32, 38, 39]. Even relatively small changes in prescribing are likely to have beneficial effects on resistance at a population level [16]. Detecting a difference in proportions between 0.70 and 0.55 at the 5% significance level and with 90% power requires a total of 434 participants, inflated to 544 to account for the loss to follow-up of approximately 20% of participants. In addition, we aim to have sufficient power to demonstrate that participants managed with CRP POCT are no worse (non-inferior), compared to those managed without CRP POCT, in terms of their COPD health status measured by the CCQ 2 weeks following randomisation. Assuming an expected difference between groups of zero, a non-inferiority margin of 0.3 lower than the lowest minimal clinically important difference and a common standard deviation of 1.1 [40], based on a one-sided significance level of 0.05 and 90% power, we would need 462 participants, inflated to 580 to account for the loss to follow-up of approximately 20% of participants.

Formulating our overall hypothesis using the Intersection-Union Test [41] we will carry out our individual subhypothesis tests at the 5% level and, if both are significant, conclude overall significance at the 5% level. Power will be affected by the level of correlation between the two outcomes and their respective effect sizes. The impact on overall power is at its greatest when there is zero correlation between outcomes and effect sizes are identical (in this case, the overall power is the product of the powers for testing each individual subhypothesis) [42, 43], and decreases with increasing correlation of outcomes and the more different effect sizes become. We do not expect our effect sizes to be similar, as our co-primary outcomes are two very different constructs (i.e. not two patient-reported outcome measures that are likely to yield similar effect sizes). We also anticipate that the outcomes will not be entirely independent (in those participants who do in fact require antibiotics, antibiotic consumption is likely to be related to COPD health status). We will, therefore, aim to recruit at least 650 participants to maintain an overall power of between 81 and 90%.

### Statistical analysis

Participant characteristics and clinical measures will be summarised using frequencies and percentages, means and standard deviations, or medians and interquartile ranges as appropriate. There will be no planned interim analysis. The assessments made for the internal pilot will be based on recruitment and follow-up rates with no between-group comparison of outcomes. All analyses will be presented as estimates of treatment effects (adjusted mean differences or odds ratios, as appropriate), with associated 95% CIs and *p* values. The main trial analysis will be based primarily on modified intention-to-treat (MITT) population, which will include all randomised participants who provide outcome data, regardless of protocol deviations or intervention received. Missing outcome data will be imputed using multiple imputation in order to obtain a secondary analysis of the primary outcomes based on the full intention-to-treat (ITT) population. A Complier Average Causal Effect (CACE) analysis, that takes into account departures from randomised treatment while maintaining a comparison of groups as randomised, will also be conducted on the primary CCQ analysis [44]. The conclusions drawn on the primary CCQ analysis will be based on both the MITT and CACE analyses. (i.e. the upper limit of the one-sided 95% CI will have to exclude 0.3 in both analyses for non-inferiority to be concluded).

All planned analyses will be described in detail in a Statistical Analysis Plan (SAP), which will be finalised prior to database lock.

### Primary analysis

Our first primary analysis will compare the odds of consuming an antibiotic for an acute exacerbation during the 4 weeks following randomisation, in each trial arm, using logistic regression. Our second primary analysis will compare the mean CCQ score between each trial arm using linear regression, with baseline CCQ scores included as a covariate, and a one-sided 95% CI constructed to assess non-inferiority. We will test if two-level models are required, due to clustering by practice, and fit a single level model if it is not needed. Modelling assumptions will be tested, with appropriate adjustments made in the presence of any violations. Missing primary outcome data are likely to be minimal, but will be accounted for in sensitivity analyses using multiple imputation, where we will assume that primary outcome data are missing at random given observed measurements.

Our second primary analysis, testing the non-inferiority of management with CRP versus no CRP with respect to the CCQ, will be based on our pre-specified margin of 0.3. Should our observed difference be between 0.3 and 0.4 (0.4 is the minimal clinically important difference (MCID) for the outcome), we will consider our results more fully, reflecting on differences found in antibiotic consumption and secondary outcomes (e.g. antibiotic resistance, EQ-5D etc.) in the two trial arms.

Further secondary analyses of the primary outcomes will be conducted:

1. To determine whether any changes in inclusion/exclusion criteria following the internal pilot had any impact on trial findings. The primary analyses will be extended by including an explanatory variable that indicates whether a participant was recruited before or after the change in criteria. The interaction between this variable and trial arm will be included to assess any differential effect this change may have had on the conclusions of the study
2. Modelling antibiotic consumption as repeated observations within individuals using a generalised linear mixed model

### Subgroup

Differential intervention effects on the primary outcomes will be assessed by fitting interaction terms in the primary models between trial arm and the following:

- COPD severity (Gold grades I/II/III/IV), from most recent spirometry assessment
- Severity of COPD exacerbation (Anthonisen criteria type 1, 2 or 3)
- Presence of a potentially pathogenic bacteria cultured from sputum sample at baseline

Two exploratory mediation analyses will be conducted using causal modelling techniques to determine whether the effect of the intervention on: (1) antibiotic prescribing and (2) COPD health status is mediated through steroid prescribing.

### Secondary analysis

Secondary outcomes will be analysed in a similar manner to the primary outcomes, with linear, logistic, and Poisson regression models fitted as appropriate.

### Economic evaluation

A within-trial health economic analysis will be undertaken from a health service perspective (UK NHS). Costs due to patient absences from work will also be considered but reported separately. The health economic evaluation will include cost-effectiveness, cost-utility and cost-consequences analyses. A trial-based budget impact analysis will be undertaken to estimate the likely impact of the use of CRP POCT in the management of antibiotic prescribing for COPD on NHS budgets. Prior to commencement of the analysis, a health economic analysis plan will be produced and reviewed by the trial team to be incorporated in the SAP.

Costs will include all resources used in the delivery of CRP POCT. This includes staff training (including travel if relevant), costs of CRP POCT kits and staff time required within the general practices. Resource use through CRP POCT implementation will be estimated through interviews with general practice staff, manufacturers and the trial team. Where additional costs are required (e.g. related to the CRP POCT test), we will obtain them from relevant sources, e.g. manufacturer list prices. Health care resource use (e.g. antibiotic prescribing and consumption, use of other COPD treatments including orally administered steroids primary- and secondary-care consultations, hospitalisations, adverse events) will be collected using data from the in-trial CRFs, participant booklet, the 4-week follow-up questionnaire and an adapted Client Service Receipt Inventory (CSRI) integrated in the 6-month note review to assess the change in profile of health care use as a result of the intervention compared to usual care. Costs will be assigned using published unit costs (e.g. PSSRU, *British National Formulary* (BNF) and NHS reference costs) where available and valued in £ sterling. The health care costs in both the intervention and the control arm will be summated with mean difference per patient in costs (including 95% CIs) calculated for the intervention compared to usual care. As the trial duration is less than 12 months, discounting will not be applied to costs or outcomes.

The primary co-outcomes (assessed at 4 weeks) will be used in the cost-effectiveness analysis. Given that the aim of the trial is whether CRP POCT-informed management of patients with AECOPD can reduce antibiotic use without negatively impacting on recovery, we will consider a range of scenarios. A base-case analysis will report an incremental cost-effectiveness ratio (ICER) presenting the additional cost of producing an extra unit (%) reduction in antibiotic prescribing (expressed as cost per unit % antibiotic prescription avoided). If the main trial fails to demonstrate non-inferiority in terms of the CCQ (as defined above) then the intervention would (if usual conditions apply) likely be regarded as not cost-effective.

We will test a range of scenarios as part of the sensitivity analysis, e.g. best case/worse case scenarios based on the results of the co-primary outcomes to explore the impact on the ICER. A threshold analysis will be undertaken to assess the willingness to accept of the costs of obtaining a reduction in antibiotic prescribing, should the CCQ score fall between the values which will warrant further exploration within the main trial analysis. In addition, we will also explore possible scenarios to reflect subsequent adoption in routine general practice, e.g. exclude the purchase and running costs of the CRP POCT equipment to reflect that the equipment may be used with general practices for a variety of POCT interventions.

We will also undertake a within-trial cost-utility analysis (CUA) to assess the incremental costs per quality-adjusted life year (QALY) gained as a result of the use of CRP POCT compared to usual care at 6 months using the EQ-5D to generate QALYs. QALYs incorporate quantity of life (additional life years) and quality of life in one measure. Thus, by dividing the difference in costs by the difference in QALYs, cost per QALY can be calculated for each comparison.

Generally, the UK National Institute for Health and Care Excellence (NICE) considers an intervention cost-effective if one of the following applies.

- The intervention is less costly and more clinically effective compared with all other relevant alternatives. In this case, no ICER is calculated as the strategy in question dominates the alternatives
- The intervention has an ICER of less than £20,000 per QALY compared to the next best alternative. This means that an investment of up to £20,000 in order to achieve an additional QALY is considered cost-effective

The ICER resulting from the CUA will be compared to the willingness-to-pay threshold of £20,000 per QALY gained as standardised by NICE. No conditions for non-inferiority will be applied in this analysis. Results will be reported as ICERs showing the extra cost of producing one extra QALY or the extra savings achieved by sacrificing one additional QALY.

For both analyses, deterministic sensitivity analysis will be undertaken to assess the extent to which parameter uncertainty affects the ICER. Probabilistic sensitivity analyses will be run to estimate the probability of the ICER falling below a range of willingness-to-pay (or accept) thresholds as standardised by NICE. For the cost-effectiveness analysis, no such threshold exists, thus we will examine the academic literature and opinions from the clinical team on what would constitute a suitable willingness to pay.

A cost-consequence analysis will present all relevant primary and secondary outcomes alongside the costs in tabular form (without combining them into ICERs) to leave decision-makers the option to form their own view of relative importance.

A trial-based budget impact analysis (BIA) will be undertaken to estimate the likely impact of the use of CRP POCT on NHS budgets through implementation costs, changes in health care usage and potential reduction of antimicrobial resistance. The BIA will be based on the size and composition of the trial population and informed by trial data supplemented by the best available published evidence where required. Sensitivity analyses will be undertaken to estimate the range of a potential budget impact considering parameter uncertainty.

### Qualitative evaluation

Interviews will be fully transcribed verbatim and checked for accuracy. Data will be analysed using framework analysis. This is a systematic approach to a thematic qualitative analysis that allows for easy comparisons between and within cases, facilitates sharing and discussion of data, and allows for clear linking/access from developed themes to original data [45]. Framework analysis involves five stages: (1) familiarisation with the data, (2) development of a thematic framework, (3) applying thematic codes to all of the data (indexing), (4) retrieving and summarising coded data in a chart and (5) interpreting the data by drawing inferences and pulling together relevant themes. Framework analysis is particularly useful when there are a number of clear research aims that have guided the questions, while allowing new themes to emerge from the data that are relevant to the research question. Dual coding will be carried out for 10% of the interviews to allow for an assessment of coding validity. NVivo qualitative analysis software will be used to assist coding.

## Discussion

AECOPD lead to increased morbidity, emergency hospital attendances, hospitalisations, health care costs, and more rapid disease progression and deterioration in quality of life [46]. Most exacerbations are managed without recourse to POCTs and most patients are prescribed antibiotics, despite evidence that these prescriptions do not always benefit them and may cause harm to the individual patients and contribute to antimicrobial resistance. POCT CRP testing may help clinicians better target antibiotic prescribing, ensuring that antibiotics are prescribed for those patients who benefit and other interventions are targeted to those who will not.

POCTs have been widely promoted for improving the care of patients who have acute infections [1]. Most evaluations of diagnostic devices consider analytic performance without evaluating impact on patient outcomes or costs. However, new tests should not be introduced into routine clinical care if they do not improve outcomes that matter to patients individually or to society, including consideration of impact on recovery and quality of life, antibiotic prescribing and resistance [2–4].

The PACE study will be the first pragmatic, randomised controlled, clinical trial to evaluate the clinical and cost-effectiveness of the addition of CRP POCT to clinical assessment for AECOPD in primary care. It will establish whether a CRP POCT can safely and cost-effectively be used to target antibiotic treatment for AECOPD so that overall antibiotic prescribing and thus consumption is decreased without compromising patients’ COPD health status. If the results of the study are positive (i.e. significant reduction in antibiotic prescribing with no evidence of significant impairment in symptomatic recovery) then implementation into practice is likely to be achieved swiftly. NICE guidelines on pneumonia already recognise a role for CRP POCT in deciding whether to prescribe antibiotics, and the use of CRP POCT is increasing in the UK. Potential barriers to uptake include the cost of testing, training, quality control and integration with laboratory systems, connectivity with electronic medical records, practitioner attitudes, and impact on clinical workflow. However, these barriers have been successfully mitigated in many European countries where CRP POCT use is now widespread [47].

### Trial status

At the time of manuscript submission, the status of this study is that patient recruitment is ongoing.

## Additional file

Additional file 1: SPIRIT 2013 Checklist: recommended items to address in a clinical trial protocol and related documents. (DOC 123 kb)

## Abbreviations

AECOPD: Acute exacerbation chronic obstructive pulmonary disease
AUC: Area under the curve
BIA: Budget impact analysis
BNF: British National Formulary
CACE: Complier Average Causal Effect
CCG: Clinical Commissioning Groups
CCQ: Clinical COPD Questionnaire
CE: European Conformity
COPD: Chronic obstructive pulmonary disease
CRF: Case Report Form
CRP: C-reactive protein
CRQ-SAS: The Chronic Respiratory Disease Questionnaire, self-administered, standardised
CSRI: Client Service Receipt Inventory
CUA: Cost-utility analysis
EQ-5D: European Quality of Life-5 dimensions
EUCAST: European Committee on Antimicrobial Susceptibility Testing
GCP: Good Clinical Practice
GOLD: The Global Initiative for Chronic Obstructive Lung Disease
GP: General practitioner
HRQoL: Health-related quality of life
ICER: Incremental cost-effectiveness ratio
ITT: Intention-to-treat
LRTI: Lower respiratory tract infection
MALDI-ToF-MS: Matrix Assisted Laser Desorption Ionising Time of Flight Mass Spectrometry
MITT: Modified intention-to-treat
NICE: National Institute for Health and Care Excellence
NIHR: National Institute for Health Research
OR: Odds ratio
PIS: Patient Information Sheet
POCT: Point-of-care test
PSSRU: Personal Social Services Research Unit
QALY: Quality-adjusted life year
RCT: Randomised controlled trial
REC: Research Ethics Committee
RR: Risk ratio
SACU: Specialist Antimicrobial Chemotherapy Unit
SAP: Statistical Analysis Plan
UK: United Kingdom
UKECA: United Kingdom Ethics Committee Authority
